# Long-term outcomes of physical activity counseling in in-patients with major depressive disorder: results from the PACINPAT randomized controlled trial

**DOI:** 10.1038/s41398-024-02885-0

**Published:** 2024-03-23

**Authors:** Jan-Niklas Kreppke, Robyn Cody, Johannes Beck, Serge Brand, Lars Donath, Anne Eckert, Oliver Faude, Martin Hatzinger, Christian Imboden, Undine E. Lang, Sebastian Ludyga, Sarah Mans, Thorsten Mikoteit, Anja Oswald, Nina Schweinfurth-Keck, Edith Holsboer-Trachsler, Lukas Zahner, Markus Gerber

**Affiliations:** 1 Department for Sport, Exercise and Health, University of Basel, Basel, Switzerland; 2 Psychiatric Clinic Sonnenhalde, Riehen, Switzerland; 3 Adult Psychiatric Clinics (UPKE), University of Basel, Basel, Switzerland; 4 Sleep Disorders Research Center, Kermanshah University of Medical Sciences, Kermanshah, 6719851115 Iran; 5 Substance Use Prevention Research Center and Sleep Disorder Research Center, Kermanshah, University of Medical Sciences (KUMS), Kermanshah, 6715847141 Iran; 6 School of Medicine, Tehran University of Medical Sciences (TUMS), Tehran, Iran; 7 German Sport University Cologne, Department of Intervention Research in Exercise Training, Cologne, Germany; 8 Psychiatric Services, Solothurn, and Medical Faculty, University of Basel, Basel, Switzerland; 9 Private Clinic Wyss, Muenchenbuchsee, Switzerland; 10 University Hospital of Psychiatry and Psychotherapy, University of Bern, Bern, Switzerland

**Keywords:** Depression, Scientific community

## Abstract

Major depressive disorder (MDD) is an increasingly common psychiatric illness associated with a high risk of insufficient physical activity, which in turn is associated with negative mental and physical health outcomes. Theory-based, individually tailored, in-person and remote physical activity counseling has the potential to increase physical activity levels in various populations. Given this, the present study investigated the effect of such a physical activity intervention on the physical activity behavior of in-patients with MDD. This was a multi-center, two-arm randomized controlled trial including initially insufficiently physically active adult in-patients with MDD from four study sites in Switzerland. The sample consisted of 220 participants (*M*_age_ = 41 ± 12.6 years, 52% women), 113 of whom were randomized to the intervention group and 107 to the control group. The main outcome, moderate-to-vigorous physical activity (MVPA), was assessed at three time points via hip-worn accelerometer. According to accelerometer measures, there was no significant difference in minutes spent in MVPA over a 12-month intervention period when comparing the intervention with the control group (*β* = −1.02, 95% CI = −10.68 to 8.64). Higher baseline physical activity significantly predicted physical activity at post and follow-up. This study showed that it is feasible to deliver an individually tailored, theory-based physical activity counseling intervention to in-patients with MDD, however yielding no significant effects on accelerometer-based MVPA levels. Further efforts are warranted to identify efficacious approaches.

Trial registration: ISRCTN, ISRCTN10469580, registered on 3rd September 2018, https://www.isrctn.com/ISRCTN10469580.

## Background

Major depressive disorder (MDD) is an increasingly common psychiatric illness, which is said to be the primary cause of burden of disease worldwide by 2030 [1]. The lifetime risk of MDD is estimated to be 15–18% among the adult population, the onset is typically gradual and the course is variable, however, frequently chronic [2]. Along with the associated impaired psychosocial functioning, this disorder was also associated with decreased quality of life, cognitive functioning and overall physical health [3, 4].

Insufficient physical activity has been associated with higher risks of MDD. As such, up to 86% of individuals with MDD do not adhere to physical activity recommendations and exhibit less physical activity (standard mean difference [SMD] = −0.25, 95% CI = −0.03 to 0.15) and more sedentary (SMD = 0.09, 95% CI = 0.01 to 0.18) behaviors compared with healthy peers [5]. In addition to the detrimental health effects of both MDD and insufficient physical activity, increased costs are a growing concern, with 28% of direct health costs attributed to depression-related physical inactivity [6].

Aerobic exercise interventions delivered to adult patients with a clinically confirmed depression diagnosis for an average of 45 min, at moderate intensity, 3 times per week, for an average of 9 weeks have resulted in overall antidepressants effects (Hedges’ *g* = −0.79, 95% CI = −1.01 to −0.57), in both outpatient and inpatient settings [7]. More specifically, large effects of exercise on depressive symptoms have been found in individuals with MDD (SMD = −1.0, 95% CI = −1.39 to −0.61) compared with insufficiently active control groups [8]. Generally, exercise-based interventions have also been found to decrease mortality rates by reducing cardiovascular risk factors, which may also be true for individuals with MDD [9, 10].

Finding ways how to increase people’s physical activity levels appeared to be a global health issue [11]. According to Hagger [12] habitual physical activity is first developed by repeating the experience in a stable context in which the activity is driven by goals and rewards. Eventually, a shift occurs towards physical activity being guided by non-conscious and automatic processes. Based on this, interventions targeting self-regulatory skills in an enabling environment are recommended [12]. Physical activity counseling based on health behavior theories offers the opportunity for such an intervention [13]. For example, the Motivation Volition (MoVo) intervention consists of three in-person counseling sessions (2 group and 1 individual), in which physical activity ideas, goals and plans are developed to foster strong goal intention. Self-concordance of the goal intention, action and coping planning skills as well as positive outcome expectancies are practiced to ultimately increase a physically active lifestyle [14]. This intervention has led to increases in self-reported physical activity levels in patients with orthopedic diseases [15], cardiac diseases [16], mental illness [17] as well as overweight/obese individuals [18] and high school students [19]. Similarly, remote interventions consisting of telephone counseling sessions have been successful in increasing levels of self-reported physical activity in insufficiently active healthy adults [20] based on selected Behavior Change Techniques (BCTs) and out-patients with MDD [21] based on motivational interviewing.

It is well evidenced that physical activity has the potential for antidepressant effects [8]. The question remains how to engage individuals in physical activity, especially those who are insufficiently physically active, as is the case in individuals with MDD [5].

In this study, we report findings from the PACINPAT (Physical Activity Counseling in In-Patients with Major Depressive Disorders) study investigating the efficacy of a theory-based, individually tailored in-person and telephone physical activity counseling intervention designed for in-patients with MDD to promote a physically active lifestyle [22]. The main outcome is objectively measured moderate-to-vigorous physical activity (MVPA). Secondary outcomes are objectively measured sedentary time, light physical activity, steps per day, average acceleration and intensity gradient, self-reported MVPA and as well depression severity. The primary hypothesis was that the intervention group would engage in more MVPA compared with the control group after the intervention phase.

## Methods

### Trial design

The PACINPAT study was a multi-center, two-arm randomized controlled trial with allocation concealment, single-blinding and intention-to-treat analysis conducted in Switzerland as a cooperation between four psychiatric clinics (two private, two public) and the University of Basel. This study was designed according to the CONSORT checklist [23, 24] and the study protocol was published before the first participant was enrolled [22].

### Setting and participants

Participants were recruited continuously from the four study sites between January 2019 and October 2021. Eligibility criteria for participation in the trial were the following: adult in-patients (18 to 64 years); MDD diagnosis according to the International Classification of Disease, 10th edition (ICD-10); Beck Depression Inventory II (BDI-II) [25] score ≥17 upon admission to in-patient treatment; insufficient physically activity prior to in-patient treatment (<150 min of MVPA/week) [26]; and sufficient spoken and written German skills. Exclusion criteria were: history of bipolar disorder type I; history of schizophrenia or schizoaffective disorder; active suicidal intent; current active alcohol or drug abuse or dependency; medical contraindication for physical activity; and significant cardiovascular, neuromuscular or endocrine disorders. Contraindications for physical activity were assessed according to ACSM guidelines [27] as well as the Physical Activity Readiness Questionnaire (PAR-Q) [28].

Clinicians screened potential participants upon admission to in-patient treatment. If the inclusion criteria were fulfilled and the patient showed interest in trial participation, they were referred to the study team. Thereupon, a member of the study team informed the patient regarding the aim of the study and emphasized the voluntary nature of trial participation and data anonymization. After obtaining written consent, the patient was enrolled in the trial.

### Procedures

#### Intervention group

The intervention consisted of two phases. The first was developed based on a health behavior theory designed to facilitate a physically active lifestyle in in-patients in a rehabilitation setting, the Motivation Volition (MoVo) model [15, 29]. It comprised two in-person physical activity counseling sessions during in-patient treatment and one remote session conducted via telephone after discharge from in-patient treatment. The counseling sessions were individually tailored to the physical activity goals and preferences resulting in a physical activity plan as well as a list of physical activity-related barriers, which was used to develop strategies to overcome them. The counseling sessions were delivered by physical activity coaches, who were exercise/sport and psychology graduates specifically trained for this intervention by the study team.

The second phase of the intervention was developed based on the Behavior Change Wheel which provides 93 distinct Behavior Change Techniques (BCTs) to encourage behavior change [30]. It comprised 26 bi-weekly remote counseling sessions individually scheduled with the participants. These sessions consisted of evidence-based BCTs proven to facilitate physical activity behavior, e.g., goal setting, feedback on behavior, review of behavior goals or action planning [31]. The physical activity coach tailored the BCTs to the individual needs of the participants. This phase of the intervention was supplemented by a mobile application and text messages. The mobile application allowed the participant and coach a common platform to plan and self-monitor physical activity as well as summarize important points discussed during the counseling sessions. The text messages were written and sent by the physical activity coaches once a week to provide the participants with feedback, reminders and information. Participants who did not have access to a smart phone or computer were encouraged to keep hand-written records of their physical activity behavior. The intervention lasted 12 months in total, whereby the intervention dose differed in three subgroups (high, low, very low). More detailed information regarding the intervention and the applied BCTs are provided in the published implementation evaluation [31], and the paper addressing the short-term outcomes of the intervention [32].

#### Control group

To ensure that the control group had the same amount of contact with the physical activity coaches during their hospital stay, the control procedure consisted of two information sessions delivered in-person during in-patient treatment by the same physical activity coaches. These sessions were neither theory-based nor individually tailored, but were based on the “Core document for Switzerland” consisting of general information on the health-enhancing benefits of regular physical activity. This document was provided and published by the Swiss Federal Office of Sport in collaboration with other national institutes (https://www.hepa.ch/de/bewegungsempfehlungen.html). During the first session the information was provided in written form, while during the second session it was summarized and presented in a short animation video. The participants in the control group then had the opportunity to discuss the content with the physical activity coach. After in-patient treatment the control group received no further procedures. Both the participants in the intervention and control groups received treatment as usual in form of the treatment regime provided by the study site, which included the possibility to access to supervised or unsupervised exercise programs.

### Data collection

Data pertaining to primary and secondary outcomes were collected at three time points from January 2019 until November 2022 at the study sites: after recruitment, approximately 2 weeks after entry to in-patient treatment (baseline); 6 weeks after discharge from in-patient treatment (post); and 12 months after discharge from in-patient treatment (follow-up).

### Accelerometry-based physical activity

We measured physical activity “objectively” with the triaxial accelerometer Actigraph wGT3x-BT (Actigraph, Shalimar, FL, USA). Evidence of the validity of these accelerometer devices has been published previously [33]. Participants were instructed to wear the device on the hip for 7 consecutive days. The sampling frequency was 60 Hertz [34]. To analyze the data, we used both cut-off based analysis with defined cut-points in counts per minute (cpm) for MVPA [33] and cut-off independent analysis of the raw data for the average measured acceleration and intensity [35].

For the cut-off based evaluation, we used an epoch length of 60 s [34]. The raw data were read into the ActiLife software V.6.13.4 (ActiLife, Shalimar, FL, USA). We used the following cut-points for assessing physical activity behavior: sedentary: <610 cpm, light: 611–2689 cpm, moderate: 2690–6166 cpm, vigorous: 6167–9642 cpm, very vigorous: >9643 cpm [33, 36]. In addition, we recorded the steps per day. For a valid measurement, participants had to wear the device for at least 4 days (including ≥ 3 valid weekdays and ≥1 valid weekend day) [37]. A day was considered valid if the device was worn for at least 8 h [38]. In addition, the participants filled in a non-wear time sheet so that we could estimate physical activity during periods when the device was not worn. If participants reported MVPA during their non-wear-time (e.g., swimming), these minutes of activity were added to the count-based activities. We classified intensities of described activities as moderate to vigorous according to the compendium of Ainsworth, Haskell [39].

We conducted the cut-off independent evaluation with the raw data using the GGIR package [40, 41]. The processing of the raw data includes an auto-calibration taking gravity into account and the calculation of the average acceleration of the gravity-averaged dynamic acceleration (Euclidean norm minus 1 g, ENMO), which is averaged over 5 s epochs in milligravitational units (mg) [42, 43]. To include the data in the analysis, participants had to wear the device for at least 4 days. The exact description of the procedure of the GGIR package has been published previously [43]. With this procedure, we were able to determine the average acceleration (AvAcc), the intensity gradient (IG), the accumulated moving time (intercept) as well as explanatory variance of the evaluation (R^2^). These values allow for comparability with other studies [35, 44].

### Self-reported physical activity

We also assessed MVPA via a self-report measure, the Simple Physical Activity Questionnaire (SIMPAQ). The SIMPAQ was developed for psychiatric patients and evidence with regard to the reliability and validity of this instrument has been published previously [45]. The average hours per day spent in bed, sitting, walking, engaging in structured sports and other unstructured activities of at least moderate intensity resulting approximately in 24 h, in the preceding 7 days were captured [45, 46]. We adjusted the evaluation slightly and additionally asked how much time was spent walking at moderate intensity. Thus, we calculated MVPA in minutes per day using moderate-intensity walking, exercise and sports activities, and other moderate-intensity activities.

### Anthropometrics

We measured body weight in kilograms (kg) with a digital weighing scale (BC-545; Tanita, USA) with a precision of 0.1 kg. Participants were weighed without shoes, wearing light clothes. We measured height in centimeters (cm) with a stadiometer with a precision of 0.5 cm. These two measurements resulted in the Body Mass Index (BMI). In addition, we measured the waist circumference with a flexible tape at the natural waist (half-way between the ribcage and the iliac crest).

### Depression severity

Depression severity was measured via self-report questionnaire using the Beck Depression Inventory-II (BDI-II) [25]. This is a reliable, validated and frequently used instrument [47, 48] containing 21 questions to asses affective, behavioral and somatic symptoms of unipolar depression (e.g., “I am so unhappy/sad that I can’t stand it”). Answers range from 0 to 3. To calculate a sum score, the values of each question are summed up. The sum score ranges between 0 and 63, with higher scores reflecting more severe depression symptoms [49, 50].

### Sample size and randomization

Previously, MoVo-based physical activity counseling has been associated with moderate effects on physical activity (*d* = 0.50) [14, 15]. Physical activity counseling in out-patients with depression has also been associated with a moderate effect (*d* = 0.45) [21]. However, remote physical activity counseling interventions including telephone and internet-based interventions have been associated with small effects (*d* = 0.20) [51]. Based on these observations, we expected a small-to-moderate effect of our physical activity counseling intervention on participants’ physical activity levels (*d* = 0.30). Using G*Power 3.1 software, this led to an estimated minimal sample size of 278 participants at follow-up (two independent groups, one-tailed, α-error probability = 0.05, power = 0.80). It was further assumed that 20% of the enrolled participants would drop out by follow-up, resulting in a target sample size of 334 participants (IG, *n* = 167; PCG, *n* = 167) [22].

Participants from whom written informed consent was obtained were randomized 1:1 into either the intervention or control group. We used a permuted block randomization with the web-based program sealed envelope (Sealed Envelope, London, UK) with the strata age, sex, and study site. Group allocation was done after the baseline data assessment by OF who was not otherwise involved in the intervention. The participants were blinded to their group allocation; however, given the nature of the trial, it was not possible to blind the study team or intervention providers.

### Data analysis

Descriptive statistics are provided as means (*M*), standard deviation (*SD*), frequencies (*n*) and percentages (%). In the case of data not being normally distributed, median (*Mdn*) and interquartile range (*IQR*) are given and marked separately. The analyses were performed on an intention-to-treat basis. Multiple imputations were performed to account for missing values. Differences in physical activity are displayed in terms of accelerometer-based minutes per day spent in sedentary behavior, light physical activity, and MVPA, as well as steps per day, average acceleration and the corresponding gradient/slope of acceleration. Additionally, minutes per day of MVPA are presented according to self-report. Differences at baseline as well as differences between the intervention and control group at post and follow-up were analyzed with linear mixed models with group, time and the interaction between group and time as fixed effects and the participant as random effects. All models were adjusted for baseline values of the dependent variable, age, sex, BMI and BDI-II upon randomization, study site and accelerometer wear time per day. The results are presented as regression coefficients with corresponding 95% confidence intervals (*CI*). Negative differences in means indicate lower scores in the control group. The model assumptions were verified using residual plots. A subgroup analysis was performed according to intervention dose using analysis of co-variance (ANCOVA) with baseline MVPA as covariate and Tukey post-hoc testing. Statistical significance was set at *p* < 0.05. The statistical analyses were performed in Stata 15 (StataCorp, College Station, Texas, USA) and Jamovi [52].

### Ethical considerations

Ethical approval for this trial was received from the Ethikkommission Nordwest- und Zentralschweiz (EKNZ; approval number 2018-00976) and from the local ethical boards of the participating study sites. The described procedures were conducted according to the ethical principles of the Declaration of Helsinki. All study participants provided written consent to participate in the study upon being informed of the aims, the voluntary nature of their participation, their right to withdraw at any time without negative consequences as well as the anonymization and publication of their data.

## Results

### Participant characteristics

**Fig. 1:**
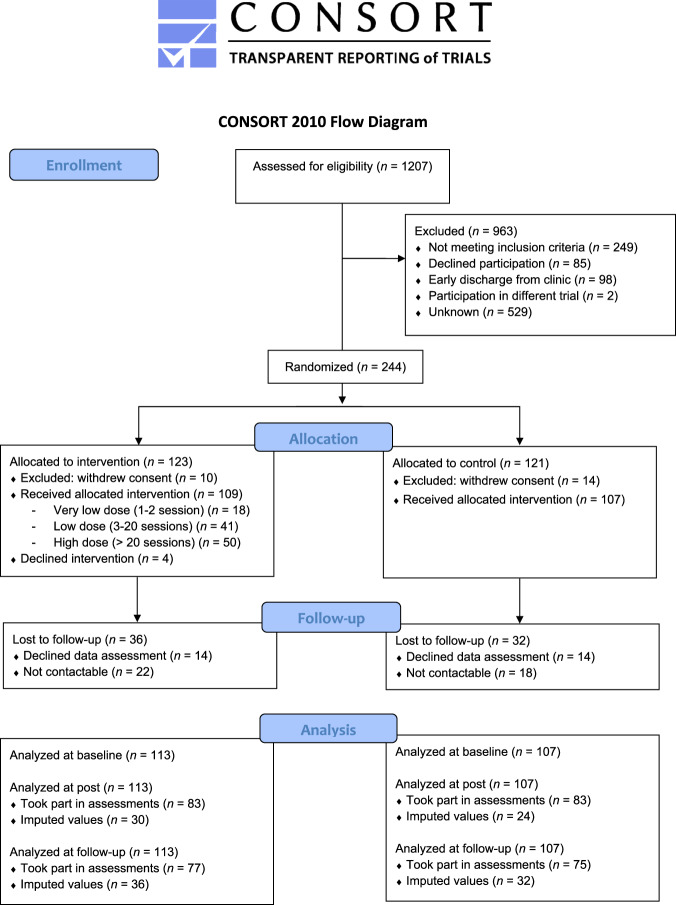
Flow diagram of the study participants. Flow diagram of the study participants according to the Consort guidelines.

The targeted sample size of 334 in-patients was not achieved because of disruption and reduced recruitment caused by the COVID-19 pandemic. Recruitment was finalized with 254 participants of which 244 had a diagnosis corresponding with the inclusion criteria. After randomization, 24 participants (10%) withdrew their consent (intervention group: *n* = 10 control group: *n* = 14) and did not take part in any data assessments or intervention. The baseline sample consisted of 220 participants (*M*_age_ = 41 years, 52% women) with 113 in the intervention group and 107 in the control group, 166 participants took part in post assessments and 152 in the follow-up assessment. In the intervention group, 50 participants (44%) received a high dose (>20 coaching sessions), 41 participants (36%) a low dose (3–20 coaching sessions) and 18 participants (16%) a very low dose (1–2 coaching sessions). In the intervention group, 4 participants (4%) were early dropouts and received no coaching sessions. The study participants are represented in a flow diagram in Fig. 1 according to the Consort guidelines.

On average, follow-up data assessments took place 55.8 weeks (*SD* = 5.9 weeks, range 47 to 95 weeks) after discharge from in-patient treatment. Thirty-six participants (32%) from the intervention group and 32 (30%) from the control group were lost to follow-up. Of those who participated in the follow-up assessments (total: *n* = 152, intervention group: *n* = 77, control group: *n* = 75), 28 participants (18%) re-entered psychiatric in-patient treatment, 31 participants (20%) were receiving partial in-patient treatment and 130 participants (86%) were in out-patient treatment during the year between discharge from in-patient treatment and follow-up assessments.

In the dropout analysis, we found no significant differences between the participants who dropped out and the participants who took part at the follow-up assessment in relation to depression severity (BDI-II), educational level (years in education), physical activity, sex and age at baseline.

More information regarding characteristics and demographic background of the participants can be found in Table 1. More information regarding primary and secondary diagnosis are available in Supplement 1 and information regarding medication in Supplement 2. Additional information on the correlations of the main variables at baseline and the correlations of the change scores from baseline to follow-up is provided in Supplements 3 and 4.

**Table 1:** Sample characteristics, separately for the intervention and control group.

	Total sample (*n* = 220)			Intervention group (*n* = 113)		Control group (*n* = 107)	
	*N*	*M*	SD	*M*	SD	*M*	SD
Age (years)	220	40.9	12.6	41.8	12.9	40.0	12.2
Height (cm)	220	171	9.6	172	10.1	171	9.0
Weight (kg)	220	80.2	21.0	79.7	22.6	80.7	19.4
Body Mass Index (BMI; kg/m^2^)	220	27.1	6.2	26.8	6.4	27.5	6.1
Waist circumference (cm)	220	90.2	17.8	89.2	17.4	91.3	18.2
Years of education^a^	218	14.3	3.3	14.2	3.5	14.5	3.2
Depression severity (BDI-II) at baseline^b^	215	22.2	10.3	23.4	11.3	21.0	9.0

	*N*	Mdn	IQR	Mdn	IQR	Mdn	IQR
Years of professional experience	220	17	22.3	18	23.5	15	19.5
Number of previous depressive episodes^c^	204	1	3	2	3.0	1	2.0
Age (years) at first depressive episode^d^	205	28	24	27.5	25	29	22
Number of antidepressants at baseline	220	1	1	1	1	1	1
	***n***	***%***	***n***	***%***	***n***	***%***	
Sex							
Female	115	52	63	56	52	49	
Male	105	48	50	44	55	51	
Nationality							
Swiss	182	83	95	84	87	81	
Foreign	38	17	18	16	20	18	
Language							
German	191	87	100	88	91	85	
Foreign	29	13	13	12	16	15	
Civil status							
Single	157	71	85	75	72	67	
Married/in a relationship	63	29	28	25	35	33	
Children living at home							
Yes	53	24	28	25	25	23	
No	167	76	85	75	82	77	
Employment							
Yes	157	71	79	70	78	73	
No	63	29	34	24	29	27	
Income^e^							
<50’000 CHF/year	84	44	43	45	41	44	
50’000 to 100’000 CHF/year	65	34	31	32	34	37	
>100’000 CHF/year	40	21	22	23	18	16	
Smoking status							
Smoking	90	41	49	43	41	38	
Non-smoking	130	59	64	57	66	62	
Active enough self-reported^f^							
Yes	51	23	25	22	26	25	
No	167	77	87	78	80	75	

*n* frequencies, *M* mean, *SD* standard deviation*, Mdn* median, *IQR* interquartile range, *cm* centimeter, *kg* kilogram, *m*^*2*^ meter squared, *BDI-II* Beck Depression Inventory-II, *CHF* Swiss franc.

^a^2 patients with missing values.

^b^5 patients with missing values.

^c^16 patients with missing values.

^d^15 patients with missing values.

^e^31 patients with missing values.

^f^2 patients with missing values.

### Physical activity and depression severity

Table 2 shows the different physical activity data at baseline for all participants and separated for the intervention and control group. At baseline, the control group had slightly more accelerometer-based and self-reported minutes spent in MVPA, slightly more light physical activity and a slightly lower intensity gradient, which represents a higher intensity. In addition, Table 3 shows the unadjusted data on different physical activity data and depression severity at the three assessment time points.

**Table 2:** Descriptive statistics of main variables at baseline, separately for the intervention and control group.

		Total sample (*n* = 220)		Intervention group (*n* = 113)		Control group (*n* = 107)	
	*n*	*M*	*SD*	*M*	*SD*	*M*	*SD*
Accelerometer-based data							
MVPA (min/day)^a^	188	57	27.2	54.5	27.3	59.9	26.9
SB (min/day)^a^	188	573	90.3	573	97.5	574	82.1
Light PA (min/day)^a^	188	175	49	170	48.4	180	49.4
Steps per day^b^	188	8060	2871	7750	2847	8405	2874
Average acceleration (mg)^c^	192	12.1	4.56	11.7	4.45	12.6	4.66
Intensity regression line^d^							
Intensity gradient	192	−3.4	0.47	−3.5	0.42	−3.3	0.50
Intercept	192	16.5	1.39	16.7	1.24	16.3	1.52
Explained variance R^2^ (%)	192	0.9	0.06	0.9	0.06	0.9	0.06
Self-reported data							
MVPA (min/day)^e^	219	42.8	48.6	39.7	41.2	46.1	55.3

*n* frequencies, *M* mean, *SD* standard deviation*,* *MVPA* moderate-to-vigorous physical activity, *SB* sedentary behavior, *PA* physical activity, *min* minutes, *mg* milligravitational units.

^a^32 patients with missing values.

^b^32 patients with missing values.

^c^28 patients with missing values.

^d^28 patients with missing values.

^e^1 patient with missing values.

**Table 3:** Unadjusted data of main variables, separately for the intervention and control group for all assessment time points.

	Intervention group						Control group					
	Baseline		Post		Follow-up		Baseline		Post		Follow-up	
	*M (SD)*	*n*	*M (SD)*	*n*	*M (SD)*	*n*	*M (SD)*	*n*	*M (SD)*	*n*	*M (SD)*	*n*
Accelerometer-based data												
MVPA (min/day)	54.5 (27.3)	99	45.8 (30.1)	72	46 (30)	61	59.9 (26.9)	89	47.3 (25.3)	72	49.7 (28.6)	55
SB (min/day)	573 (97.5)	99	581 (171)	72	543 (125)	61	574 (82.1)	89	525 (90.3)	72	556 (143)	55
Light PA (min/day)	170 (48.4)	99	198 (68.8)	72	214 (95.6)	61	180 (49.4)	89	192 (75.5)	72	215 (74.4)	55
Steps per day	7750 (2847)	99	6999 (3452)	72	7056 (3550)	61	8405 (2874)	89	6749 (2860)	72	7326 (2892)	55
Average acceleration (mg)	11.7 (4.45)	101	11.4 (4.38)	71	11.5 (4.12)	62	12.6 (4.66)	91	12.1 (4.28)	74	12.7 (4.11)	56
Intensity regression line												
Intensity gradient	−3.5 (0.42)	101	−3.6 (0.5)	71	−3.4 (0.52)	62	−3.3 (0.5)	91	−3.3 (0.53)	74	−3.44 (0.57)	56
Intercept	16.7 (1.24)	101	16.9 (1.45)	71	16.3 (1.54)	62	16.3 (1.52)	91	16.1 (1.58)	74	16.6 (1.87)	56
Explained variance R^2^ (%)	0.9 (0.06)	101	0.9 (0.06)	71	0.9 (0.05)	62	0.9 (0.06)	91	0.9 (0.06)	74	0.9 (0.05)	56
Self-reported data												
MVPA (min/day)	39.7 (41.2)	112	79.2 (88.2)	82	74.4 (91.8)	75	46.1 (55.3)	107	61.2 (78.6)	83	78 (84)	71
Depression severity												
BDI-II scores	23.4 (11.3)	109	17.4 (11.6)	81	15.2 (12.4)	67	21 (9)	106	14.6 (10.4)	83	12.9 (9.6)	69

*n* frequencies, *M* mean, *SD* standard deviation, *MVPA* moderate-to-vigorous physical activity, *SB* sedentary behavior, *PA* physical activity, *min* minutes, *mg* milligravitational units, *BDI-II* Beck Depression Inventory-II.

**Fig. 2:**
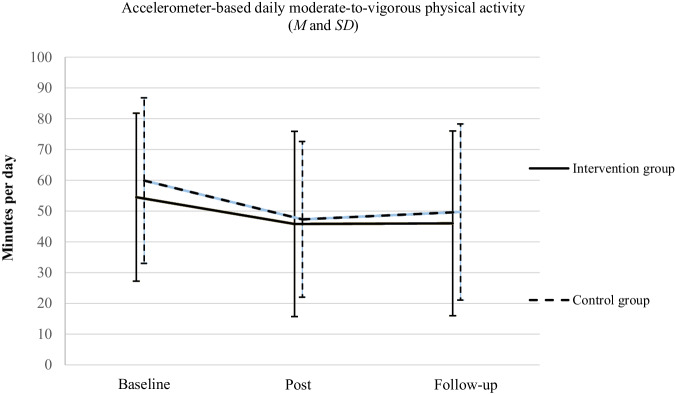
Accelerometer-based daily moderate-to-vigorous physical activity. Means (M) and standard deviation (SD) as error bars of moderate-to-vigorous physical activity (minutes/day), separately for the intervention and control group (baseline: *n* = 188, post: *n* = 144, follow-up: *n* = 116).

Figure 2 shows the device-based MVPA values at baseline, post and follow-up for the intervention and control group as means with standard deviations. It can be seen that both groups had lower MVPA values at post and follow-up than at baseline. Minutes spent in MVPA at follow-up were similar to those measured at post in both groups.

Table 4 shows group differences at post and follow-up as well as the effect of baseline values on post and follow-up values. It shows that across the groups, those who were 1 min more physically active at baseline, spend 0.4 min (95% *CI* = 0.2 to 0.6) more performing MVPA at post on average. The same conclusions can be drawn for sedentary behavior, light physical activity, steps per day, average acceleration and intensity. These effects are also seen at follow-up. Those who were one minute more physically active at baseline were 0.3 minutes more active at follow-up (95% *CI* = 0.2 to 0.5). This effect is also present for sedentary behavior, light physical activity, steps per day and average acceleration. Those who had higher average values at baseline also had higher average values at post and follow-up, when controlling for age, sex, BMI, BDI-II at baseline, clinic and accelerometer wear-time.

**Table 4:** Differences in physical activity levels and depression severity at baseline, post and follow-up.

	Overall effect Baseline to post	Overall effect Baseline to follow-up	Between group difference Post	Between group difference Follow-up
	β (95% *CI*)	β (95% *CI*)	β (95% *CI*)	β (95% *CI*)
Accelerometer-based data				
MVPA (min/day)	0.40 (0.23 to 0.58)	0.34 (0.15 to 0.54)	−1.05 (−9.72 to 7.62)	−1.02 (−10.68 to 8.64)
SB (min/day)	0.21 (0.08 to 0.35)	0.24 (0.10 to 0.39)	9.21 (−13.70 to 32.12)	7.31 (−15.19 to 29.82)
Light PA (min/day)	0.64 (0.41 to 0.86)	0.62 (0.38 to 0.86)	5.46 (−13.78 to 24.71)	3.35 (−16.08 to 22.79)
Steps per day	0.48 (0.32 to 0.64)	0.36 (0.20 to 0.52)	257 (−610 to 1124)	163 (−758 to 1084)
Acceleration	0.39 (0.24 to 0.54)	0.23 (0.08 to 0.38)	−0.68 (−1.90 to 0.55)	−0.52 (−1.82 to .78)
Gradient/slope	0.29 (0.11 to 0.46)	0.16 (-0.02 to 0.34)	−0.14 (−0.30 to 0.01)	0.05 (−0.11 to 0.21)
Self-reported data				
MVPA (min/day)	0.11 (−0.22 to 0.44)	0.13 (−0.24 to 0.49)	18.70 (−5.90 to 43.30)	−6.27 (−33.16 to 20.62)
Depression severity				
BDI-II scores	0.65 (0.45 to 0.82)	0.58 (0.39 to 0.76)	1.11 (−1.66 to 3.88)	1.58 (−1.41 to 4.58)

Results from linear mixed models, β regression coefficient representing estimated group mean difference.

*CI* confidence interval, *SB* sedentary behavior, *min* minutes, *PA* physical activity, *MVPA* moderate-to-vigorous physical activity.

The group differences show that at post and follow-up, participants of the intervention group engaged in slightly more sedentary behavior, light physical activity and steps per day, whereas they accumulated slightly less MVPA and had lower scores for average acceleration and intensity. Contrarily, the intervention group self-reported more minutes spent in MVPA at post and lower levels at follow-up. Nevertheless, all estimated values by the linear mixed models for the group differences between the intervention group and the control group at post and follow-up were not significantly different, as reflected in the reported confidence intervals.

**Fig. 3:**
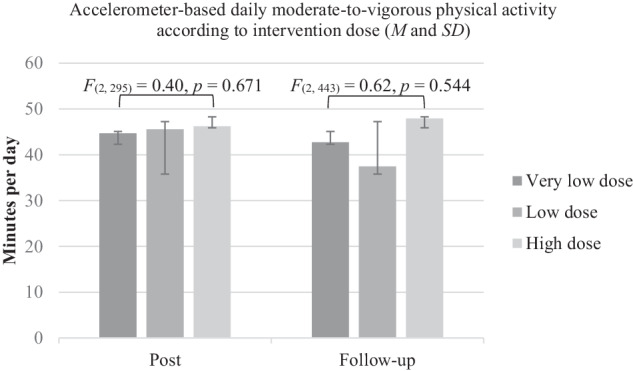
Results from Subgroup analysis using ANCOVA. Means (M) and standard deviation (SD) as error bars of moderate-to-vigorous physical activity (minutes/day), separately for very low, low and high doses at post and follow up.

As shown in Fig. 3, the subgroup analysis using ANCOVA showed that MVPA at post and follow-up did not differ between participants who received a very low, low or high dose of the counseling sessions.

## Discussion

The aim of this study was to report the key findings from the PACINPAT trial investigating the efficacy of a theory-based, individually tailored in-person and telephone-based physical activity counseling intervention designed for in-patients with MDD to promote a physically active lifestyle.

There was no evidence to support the primary hypotheses. The results of our study showed that against expectations, participants from the intervention group did not significantly differ in minutes of MVPA per day compared to participants from the control group over a 12-month intervention period. These results add to the current body of research and represent a valuable contribution to the evidence regarding the promotion of physical activity in patients with MDD. Namely that, a theory-based physical activity counseling approach might not necessarily improve physical behavior, underlining that it is challenging to overcome the issue of insufficient physical activity in individuals with MDD.

### Physical activity levels

Given the relatively high baseline levels of MVPA observed in our sample, the present study seems to corroborate that Swiss clinics are successful in integrating exercise, sport and other physical activity opportunities in their clinical structures [53], which is in line with international recommendations [54]. However, this study also shows that the level of physical activity drops once patients leave the hospital and return to their own natural life circumstances.

These results diverge from previous findings on physical activity counseling interventions. For instance, a prior physical activity counseling intervention based on BCTs delivered remotely to insufficiently physically active (healthy) adults showed a statistically significant increase in accelerometer-based MVPA in the intervention compared to the control group over a 6-month period [20]. Conversely, the results from our study showed no statistically significant difference in accelerometer-based MVPA when comparing both groups in the short or long-term, whereas self-reported MVPA increased in both groups. Increased levels of self-reported MVPA were also found in a study population of out-patients with MDD, however, significantly more so in the participants receiving physical activity counseling compared to those receiving only treatment as usual [21]. Possible reasons for these diverging outcomes could be the different study populations (in-patients vs. out-patients), differences in the study design or intervention implementation. For instance, Chalder, Wiles [21] were able to recruit a larger sample (361 patients), used motivational interviewing and applied an additional face-to-face meeting later during the intervention phase.

We observed non-significant differences in self-reported MVPA between the groups at both post and follow-up. According to previous research varying physical activity self-reporting in individuals with MDD is a common phenomenon [5]. Generally, over or under-reporting of physical activity may be related to many factors such as cognition (lower cognitive function associated with over-reporting) [55], differences in the perception of physical exertion [56] and wearing a measurement device (which reveals no effect on device-measured MVPA but may lead to an over-reporting of self-reported MVPA) [57]. Furthermore, we did note a positive, yet not significant, correlation between device-measured and self-reported MVPA. This finding is in line with prior results of a systematic review stating that correlations between self-reported and direct measures (including accelerometer) of physical activity may range between -0.71 and 0.96 [58]. More recent research supports this, stating that the agreement between objective and self-reported physical activity is low (ranging from ĸ = 0.07 (95% *CI* = 0.02 to 0.12) to 0.19 (95% *CI* = 0.13 to 0.24]) [59].

While symptom severity was comparable in both studies, the fact that the first data assessment took place during in-patient treatment (and not in natural life circumstances) may have complicated the detection of increased physical activity levels in our trial. Nevertheless, our correlation data show that at baseline the depression severity is inversely correlated with the accelerometer-based data, as has already been confirmed by other authors [60]. However, in contrast to previous studies [61], the change scores of the depression severity and the physical activity data show no correlation and the points mentioned do not explain why we did not observe any significant time by group effects in our population. One possible explanation is that we used a placebo control group which also received some basic form of physical activity counseling. Another explanation might be that out-patients respond better to remote physical activity counseling. Previous studies have also shown that programs, in which patients meet the coach more often, and practice exercise and sport activities together with the coach have positive effects [62, 63], a finding which was also observed in treatment-resistant patients with depression [64]. As we have highlighted in our qualitative analysis, participating in a physical activity counseling program may put pressure on some patients who reported suffering from the feeling of failing at activities in the past and concerns that participating in such a program may represent a potential for experienced failure in the future [65]. Therefore, closer accompaniment at the beginning of the intervention might be helpful to counteract this pressure and to optimize the efficacy of physical activity counseling in in-patients.

### Physical activity counseling

As shown in our study, physical activity counseling in in-patients with MDD remains a challenge [66]. An important phenomenon may be individual patient trajectories characterized, among other factors, by illness experience and mindset towards physical activity, influencing motivation [65], which in turn may influence self-regulation of physical activity behavior [67]. This may explain the high dropout rate in the present study and the fact that physical activity remained nearly unchanged after discharge from the clinic. In the case of lacking evidence for an intervention effect, alternative explanations are possible to account for the non-significant differences. For instance, it is possible that the intervention was effective, but insufficiently implemented. Alternatively, it could be that the intervention in fact was not effective [68]. According to our implementation evaluation, the intervention reached the intended population in varying doses, and the fidelity was continuously monitored and predominantly achieved with few adaptations which were well-documented [31]. More specifically, 16% of the participants receiving the intervention dropped out after only two counseling sessions, whereas 36% received up to 75% of the intended intervention dose and 44% received 75% or more of the intended dose. However, additional analyses showed that participants who received low and high intervention doses had similar profiles with regard to objectively measured MVPA across the intervention period. In addition, we acknowledge that due to the COVID-19 pandemic, we were not able to recruit the intended minimal sample size of 334 participants. With regard to the intervention content, fidelity to the underlying theory was given according to documentation by our trained intervention implementers (coaches). More specifically, BCTs evidenced to improve physical activity behavior according to a meta-analyses [69, 70] were indeed implemented [31]. Furthermore, there is evidence suggesting that the intervention may have been effective for only a sub-population of the study participants. According to a qualitative analysis of the PACINPAT trial, the intervention was experienced in four distinguishable ways with only one (expansive experience) leading to increased wellbeing and maintained physical activity levels [65]. In this sense, the heterogeneity of experiences of the individuals in the study may have contributed to the absence of an intervention effect.

In the case of the intervention itself being ineffective, meta-analytic data of RCTs suggest that interventions based on socio-cognitive theories to increase physical activity overall show small effect sizes (standard mean differences from 0.2 to 0.3), and these may even be overestimated due to methodological weaknesses [71]. Hence, interventions solely relying on socio-cognitive theories may in fact not be the best approach. Increasing evidence suggests that affective responses to exercise activities have been neglected so far in cognition-based approaches (i.e., theory-based approaches) to promote physical activity [72]. It is plausible that our limited focus on affective components may have been particularly unfortunate in a population of participants with an affective disorder, often characterized by anhedonia [73]. Evidence suggests that people with MDD do feel a favorable affective response following physical activity [74]. Along these lines, the PACINPAT intervention was tailored specifically to factors pertaining to physical activity (e.g., physical activity goals and preferences), however, there was a lack of tailoring to disease-specific factors, such as affective state and disease experience [65]. The systematic review by Thomas, Thirlaway [75] confirmed that interventions involving physical activity in combination with a psychotherapeutic approach can have positive effects on the increase in physical activity levels and on psychological outcomes. Furthermore, highly personalized lifestyle medicine has been implemented in a patient with cardiovascular disease with very positive results, such as weight loss, reduction in medication and improvement in fitness [76]. Hence, this approach may also be recommended for people with other chronic diseases such MDD. This may also include applying a multidisciplinary approach, which has been evidenced to significantly improve total activity and increase MVPA in in-patients with severe mental illness [77].

### Practical implications

In future interventions for patients with MDD, it may be beneficial to focus more strongly on affective responses to exercise in addition to a theory-based cognitive approach [72, 78]. This may entail more in-person contact during the initial phase counseling sessions, allowing the coach and participant to try activities together to identify a facilitating situational framework for regular physical activity as well as activities that lead to positive affective responses. A focus on cognitive factors – as implemented in this study – can follow after, especially because the implementation evaluation revealed that most of the patients who completed the intervention were satisfied with the coach (86%) and would recommend it to other patients (63%) [31]. An alternative solution could be a group-based approach. Both individual and group sessions are known to be effective in the promotion of physical activity [79], hence, a combination of both could be beneficial. While group exercise can increase affective responses [80], individual sessions can be optimally tailored [81]. Furthermore, in individuals with MDD, it was shown that individually prescribed physical activity can be as effective as physical activity treatment with integrated psychological techniques. The social interaction and reinforcement could mediate the positive effects of increasing physical activity levels appears to be important. According to the authors, symptom reduction is then unrelated to an individual or group setting [75]. In addition, multidisciplinary approaches could be considered to promote health in different domains [77]. This approach has been used in transdiagnostic patients, resulting in the effective treatment of different mental illnesses patterns as well as the promotion of physical activity [62].

### Methodological considerations

Within the PACINPAT study, there was a risk for selection bias, e.g., participants who were willing to engage in the trial were inherently more interested in physical activity and the promotion thereof. This means that these results cannot be generalized across all in-patients with MDD. However, according to the drop-out analyses, there were no significant differences between those participants who took part and those who were lost to follow-up. A further potential source of bias lies in the applied self-report measures, which are prone to recall and social desirability bias. However, this issue was counteracted by collecting accelerometer-based physical activity data. Accelerometer-based physical activity was measured during a period of seven days, which is considered to be representative of physical activity behavior [82]. Additionally, there is evidence stating that wearing an accelerometer does not increase accelerometer-based MVPA [57]. Despite this, it is possible that a salience effect may be present. It is worthwhile to consider whether measuring physical activity over a prolonged period of time (e.g., via smartwatches) would improve measurement, especially given that physical activity is a complex behavior with known fluctuations. For cost and practicality reasons this may be a challenge in future research. Another limitation was that we adjusted the analysis of the accelerometer data in the interest of not losing an excessive amount of data. For a valid measure, 4 days were required (as opposed to 5 days or more) and 8 h per day (as opposed to 10 h). However, evidence shows that 4 days are representative of 1 week [37, 83], and that there are no meaningful differences between 8, 10 and 12 h of wear time [38]. Hence, our approach led to the consideration of more data without compromising reliability and validity of the measure [82]. Moreover, the follow-up assessment was performed directly after the completion of the intervention, which means that we do not know how physical activity behavior developed after completion of the counseling intervention. We also acknowledge that participants were not only heterogeneous with regard to the experience and responses to the intervention, they also varied with regard to medical and psychotherapeutic treatments provided by the clinics and had varying durations of in-patient treatment. However, these effects should not have a bearing on the results because of the randomized study design. Finally, it could be that the Covid-19 pandemic may have affected the intervention. However, we were able to show that participants included before vs. after the outbreak of the pandemic did neither differ with regard to psychopathology nor physical activity behavior [84].

## Conclusion

Individually-tailored physical activity counseling drawing on socio-cognitive theory and targeting the improvement of various behavior change techniques (BCTs) was feasible in patients with MDD, but did not increase their daily MVPA. Given the challenge of long-term adherence to recommended MVPA levels, researchers are encouraged to intensify their efforts into the development of further and more efficacious approaches specifically targeted towards this patient population. Inclusion of more affect-oriented components or personalized lifestyle medicine could be needed to better support patients with MDD on their way to establishing a sustainably physically active lifestyle.

### Supplementary information

Supplement 1. Primary and secondary diagnoses to baseline

Supplement 2. Medication to baseline

Supplement 3. Correlation coefficients of main variables for baseline

Supplement 4. Correlation coefficients of change scores of main variables for baseline to follow-up

## Data Availability

The data and materials will be made available by the authors upon request, without undue reservation.
