# Supplementary material for: Long-term outcomes of physical activity counseling in in-patients with major depressive disorder: results from the PACINPAT randomized controlled trial
Source: Transl Psychiatry. 2024 Mar 23;14:160. doi: 10.1038/s41398-024-02885-0 (PMC10960795; doi:10.1038/s41398-024-02885-0)
Supplement: Supplementary file 1 — Supplement 1. Primary and secondary diagnoses to baseline [file 41398_2024_2885_MOESM1_ESM.docx]

**Supplement 1.** Primary and secondary diagnoses to baseline

|  | *n* | *%* |
| --- | --- | --- |
| *Primary diagnosis* |  |  |
| Bipolar affective disorder, currently light or moderate depressive episode (F31.3) | 2 | 1 |
| Bipolar affective disorder, currently severe depressive episode without psychotic symptoms (F31.4) | 1 | 1 |
| Moderate depressive episode (F32.1) | 46 | 21 |
| Severe depressive episode without psychotic symptoms (F32.2) | 29 | 14 |
| Severe depressive episode with psychotic symptoms (F32.3) | 1 | 1 |
| Recurrent depression, current moderate episode (F33.1) | 88 | 41 |
| Recurrent depression, current severe episode without psychotic symptoms (F33.2) | 47 | 22 |
| Recurrent depression, currently remitted (F33.3) | 1 | 1 |
|  |  |  |
| *Secondary diagnoses^a^* |  |  |
| Mental and behavioral disorders due to psychoactive substance use (F10-19) | 36 | 17 |
| Schizophrenia, schizotypal and delusional disorders (F20-29) | 0 | 0 |
| Neurotic, stress-related and somatoform disorders (F40-48) | 55 | 26 |
| Behavioral syndromes associated with physiological disturbances and physical factors (F50-59) | 13 | 6 |
| Disorders of adult personality and behavior (F60-69) | 28 | 13 |
| Mental retardation (F70-79) | 1 | 1 |
| Disorders of psychological development (F80-89) | 6 | 3 |
| Behavioral and emotional disorders with onset usually occurring in childhood and adolescence (F90-98) | 25 | 12 |
|  |  |  |
| *Somatic disorder* |  |  |
| Yes | 119 | 55 |
| No | 96 | 45 |

Notes: ^a^Multiple secondary diagnoses are possible. Accumulated percentage is therefore not equal to 100.
