# Supplementary material for: Long-term outcomes of physical activity counseling in in-patients with major depressive disorder: results from the PACINPAT randomized controlled trial
Source: Transl Psychiatry. 2024 Mar 23;14:160. doi: 10.1038/s41398-024-02885-0 (PMC10960795; doi:10.1038/s41398-024-02885-0)
Supplement: Supplementary file 2 — Supplement 2. Medication to baseline [file 41398_2024_2885_MOESM2_ESM.docx]

**Supplement 2**. Medication to baseline

|  | *n* | % |
| --- | --- | --- |
| *Antidepressant medication* |  |  |
| Selective serotonin reuptake inhibitors (SSRI) |  |  |
| Citalopram | 6 | 3 |
| Escitalopram | 41 | 19 |
| Fluoxetine | 10 | 5 |
| Paroxetine | 2 | 1 |
| Sertraline | 14 | 6 |
| Selective serotonin and noradrenaline reuptake inhibitors (SSNRI) |  |  |
| Duloxetine | 27 | 12 |
| Venlafaxine | 22 | 10 |
| Tricyclic |  |  |
| Amitriptyline | 2 | 1 |
| Clomipramine | 1 | 0 |
| Trimipramine | 3 | 1 |
| Bupropion | 25 | 12 |
| St. John’s worth extract | 3 | 1 |
| Lithium | 13 | 6 |
| Mianserin | 1 | 0 |
| Mirtazapine | 20 | 9 |
| Trazodone | 52 | 24 |
| Vortioxetine | 36 | 17 |
|  |  |  |
| *Other psychotropic medication* |  |  |
| Antiepileptics | 14 | 6 |
| Antipsychotics | 58 | 27 |
| Sedatives | 34 | 16 |
| Anxiolytics | 24 | 11 |
| Psychostimulants | 16 | 7 |
|  |  |  |
| *Other medication* |  |  |
| Antihypertensives | 32 | 15 |
| Beta blockers | 13 | 6 |
| Analgesics | 17 | 7 |
| Antacids | 26 | 12 |
| Statins | 12 | 5 |
| Antidiabetics | 6 | 3 |
| Others^a^ | 107 | 48 |

Notes: ^a^Multiple other medications are possible. Accumulated percentage is therefore not equal to 100.
