# Supplementary material for: Long-term outcomes of physical activity counseling in in-patients with major depressive disorder: results from the PACINPAT randomized controlled trial
Source: Transl Psychiatry. 2024 Mar 23;14:160. doi: 10.1038/s41398-024-02885-0 (PMC10960795; doi:10.1038/s41398-024-02885-0)
Supplement: Supplementary file 3 — Supplement 3. Correlation coefficients of main variables for baseline [file 41398_2024_2885_MOESM3_ESM.docx]

**Supplement 3.** Correlation coefficients of main variables for baseline

|  | **Accelerometer-based**  MVPA (min/day) | | | **Self-reported**  MVPA (min/day) | | | **Accelerometer-based**  Average Acceleration (mg) | | | **Accelerometer-based**  Steps per day | | |
| --- | --- | --- | --- | --- | --- | --- | --- | --- | --- | --- | --- | --- |
|  | *n* | *r* | *p* | *n* | *r* | *p* | *n* | *r* | *p* | *n* | *r* | *p* |
| **Self-reported**  MVPA (min/day) | 187 | 0.12 | 0.11 |  |  |  |  |  |  |  |  |  |
|  |  |  |  |  |  |  |  |  |  |  |  |  |
| **Depression severity** BDI-II scores | 183 | -0.17* | 0.03 | 214 | -0.02 | 0.8 | 200 | -0.14* | 0.04 | 181 | -0.21** | 0.01 |

Notes: Correlation coefficients are controlled for baseline values sex, age, BMI. *n,* frequencies; *r, Pearson`s r;* MVPA; moderate-to-vigorous physical activity; PA, physical activity; min, minutes; mg, milligravitational units; BDI-II, Beck Depression Inventory-II, * *p* < .05, ** *p* < .01
