# Supplementary material for: Long-term outcomes of physical activity counseling in in-patients with major depressive disorder: results from the PACINPAT randomized controlled trial
Source: Transl Psychiatry. 2024 Mar 23;14:160. doi: 10.1038/s41398-024-02885-0 (PMC10960795; doi:10.1038/s41398-024-02885-0)
Supplement: Supplementary file 4 — Supplement 4. Correlation coefficients of change scores of main variables for baseline to follow-up [file 41398_2024_2885_MOESM4_ESM.docx]

**Supplement 4.** Correlation coefficients of change scores of main variables for baseline to follow-up

|  | **Change score accelerometer-based**  MVPA (min/day) | | | **Change score**  **self-reported**  MVPA (min/day) | | | **Change score accelerometer-based**  Average Acceleration (mg) | | | **Change score accelerometer-based**  Steps per day | | |
| --- | --- | --- | --- | --- | --- | --- | --- | --- | --- | --- | --- | --- |
|  | *n* | *r* | *p* | *n* | *r* | *p* | *n* | *r* | *p* | *n* | *r* | *p* |
| **Change score**  **self-reported**  MVPA (min/day) | 107 | 0.142 | 0.149 |  |  |  |  |  |  |  |  |  |
|  |  |  |  |  |  |  |  |  |  |  |  |  |
| **Change score depression severity** BDI-II scores | 107 | 0.059 | 0.553 | 134 | -0.017 | 0.847 | 119 | -0.012 | 0.895 | 107 | -0.036 | 0.717 |

Notes: Correlation coefficients are controlled for baseline values sex, age, BMI. *n,* frequencies; *r, Pearson`s r;* MVPA; moderate-to-vigorous physical activity; PA, physical activity; min, minutes; mg, milligravitational units; BDI-II, Beck Depression Inventory-II, * *p* < .05, ** *p* < .01
